# Design of PI3K-mTOR Dual Inhibitors for Ovarian Cancer: Are we on the Right Track?

**DOI:** 10.2174/0109298673293028240326051835

**Published:** 2024-04-04

**Authors:** Mangala Shenoy K., Ekta Rathi, Karthik S. Udupa, Shama Prasada K., K. Sreedhara Ranganath Pai, Suvarna Ganesh Kini

**Affiliations:** 1 Department of Pharmaceutical Chemistry, Manipal College of Pharmaceutical Sciences, Manipal Academy of Higher Education (MAHE), Manipal, Karnataka, 576104, India;; 2 Department of Medical Oncology, Kasturba Medical College, Manipal Academy of Higher Education (MAHE), Manipal, Karnataka, 576104, India;; 3 Department of Cell and Molecular Biology, Manipal School of Life Sciences, Manipal Academy of Higher Education (MAHE), Manipal, Karnataka, 576104, India;; 4 Department of Pharmacology, Manipal College of Pharmaceutical Sciences, Manipal Academy of Higher Education (MAHE), Manipal, Karnataka, 576104, India

**Keywords:** Ovarian cancer, PI3K/AKT/mTOR pathway, dual inhibitors, triazine, breast cancer, fallopian tube

## Abstract

Ovarian cancer is one of the most familiar kinds of gynecological cancer seen in women. Though it is not as familiar as breast cancer, the survival rate for ovarian cancer is very low when compared with breast cancer. Even after being one among the familiar types, to date, there are no proper treatments available for ovarian cancer. All the treatments that are present currently show a high rate of recurrence after the treatment. Therefore, treating this silent killer from the roots is the need of the hour. PI3K/AKT/mTOR pathway is one of the pathways that get altered during ovarian cancer. Studies are already going on for the inhibition of PI3K and mTOR separately. Efforts have been made to inhibit either PI3K or mTOR separately earlier. However, due to its side effects and resistance to the treatments available, current studies are based on the inhibition of PI3K and mTOR together. Inhibition of PI3K and mTOR simultaneously reduces the chances of negative feedback, thus decreasing the toxicity. This review contains the evolution of PI3K and mTOR drugs that are approved by the FDA and are in the trials for different cancer types, including ovarian cancer. In this article, how a molecular targeted therapy can be made successful and free from toxicity for treating ovarian cancer is discussed. Therefore, this review paves the way for finding an effective scaffold rather than the clinical part. The scaffold thus selected can be further modified and synthesized in the future as dual PI3K/mTOR inhibitors specifically for OC.

## 1 INTRODUCTION

**Fig. (1):**
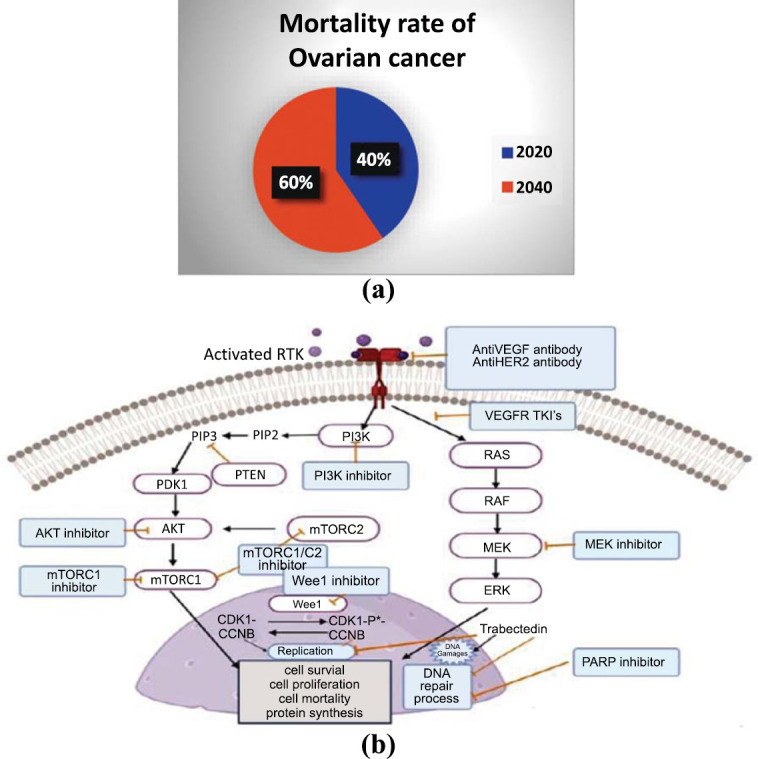
(**a**) Pie chart showing the mortality rate of ovarian cancer. (**b**) Schematic representation of pathways of ovarian cancer.

Ovarian Cancer (OC) is one of the most lethal gynecological diseases, with more than 125,000 women dying every year worldwide [1]. This count has been predicted to rise by 67% by the year 2035. According to the Globocan’s 2020 report, the number of women around the world diagnosed with OC will rise almost to 42%, *i.e.*, 445721, by 2040 [2, 3]. Besides, the mortality rate of women will upsurge by 60% (~305848 cases) in comparison with the 2020 report (207252) (Fig. **1a**) [4, 5]. Therefore, it is crucial to overcome the high incidence and mortality rate of OC in women. A comprehensive knowledge of OC is critical to determine the potent therapy. Hence, the first section has briefly overlaid the characteristics of OC and its associated pathways.

Ovarian cancer refers to the growth of the cells that form in the ovaries. It is not a single disease but an umbrella term for almost 30 types of cancer [6]. They are widely classified into 4 groups: epithelial tumor, germ cell carcinoma, stromal carcinoma, and small cell carcinoma [6]. Epithelial OC, as the name says, originates from the surface where the cells are formed in the tissues covering the ovary and fallopian tube, which constitutes 90% of the total OC. Germ cell carcinoma, which forms in the reproductive cells of ovaries and stromal carcinoma and is found in the connective tissue, are the rare types of OC. However, the extremely rare one is the small cell carcinoma. In small cell carcinoma, it is not certain if the cells are from epithelial or sex cord origin. Risk of Ovarian cancer can be seen mostly in women over the age of 50 [7]. Thus, it concludes that the risk of ovarian cancer increases after menopause. Reasons for the same could be the increased exposure to the hormone estrogen and/or higher number of ovulations as per literature [8]. OC often has no symptoms in the initial stages, but there may be chances of some common symptoms like bloating, fatigue, abdominal pain *etc.*, which are often neglected. Therefore, it is termed a “silent killer” [9]. So now the main question that arises is, “Is there any treatment for this silent killer?” Basically, chemotherapy and surgery are the two main modes of treatment for OC. During surgery, the uterus, fallopian tube, and ovaries are removed. In some cases, the debulking of cancer from the abdomen and the pelvis is also required. However, in most of the cases the recurrence of the OC was noted even after the surgery [10, 11]. Even in some cases, it is unclear to know how long the person will live after surgery. From the reports of 2019 conducted in the United States and Asia, one can find that the surgery did not improve the lifespan of people. Looking into the reasons for the same, one can find that this may be due to the improper removal of cancer during surgery. Therefore, the role of surgery in treating OC itself is questionable. Usually, the standard treatment that is prescribed for the OC is cytoreductive surgery. Platinum-based chemotherapy follows this surgery. However, it was noticed that the patients who come to the treatment develop platinum sensitivity initially, but over time, develop platinum resistance. As a result, this reduced the response rate for the next cycle of chemotherapy [12, 13]. For chemotherapy, standard drugs like carboplatin and paclitaxel are used. This is usually done during stage III or IV. However, the use of these drugs may cause some side effects like rashes, vomiting, damage of kidney, nerve damage *etc.* Therefore, the more potent drugs with minimal side effects are required to save the life of the women from the “silent killer”. Currently, researchers are exploring diverse targets worldwide to overcome the mortality rate associated with OC. As per the literature survey, mutations have been identified in PARP, BRCA, PI3K/AKT/ mTOR, HNF-1-beta and other pathways. Out of all these pathways, the PI3K/AKT/mTOR pathway is the most altered pathway in ovarian cancer cases (Fig. **1b**). Also, the OC can be classified as a heterogenous disease, therefore, molecular targeted therapy can be used to get fewer toxic effects and more positive results in the treatment [14]. So, this review is based on those drugs that are clinically approved and are in trials that target PI3K and mTOR individually as well as together and their importance in OC. This review shows the way PI3K and mTOR inhibitors were derived and how the use of different scaffolds improved their inhibition activity in treating OC. Keeping this in mind, one can design an effective scaffold to treat OC. We can see that the addition of different substitutions at different places of the scaffold brought a huge change in the activity. As a result, this article indicates the currently available targets of different cancers along with OC and the inhibitors that were designed to face undesired activity, leading to finding an alternative effective solution for an optimization.

## 2 PI3K/AKT/mTOR PATHWAY

Phosphoinositide 3-kinases (PI3K) are a class of lipid kinases. They are the major downstream effectors of the G protein-coupled receptors and receptor tyrosine kinases. Based on the structure and specifications in the substrate, PI3K further consists of 3 classes, namely, class I, class II, and class III [15]. Class I of the PI3K is comprised of the class IA and class IB PI3K’s. Class IA is heterodimers consisting of PI3K α, β and δ, which are composed of the p85 regulatory subunit and p110 catalytic subunit [16]. Class IB consists of PI3K γ. Class II of PI3K comprises PI3K-C2α, PI3K-C2β, and PI3K-C2γ isoforms. Class III comprises VPS34 and VPS15 catalytic and regulatory subunits. PI3Kα and PI3Kβ isoforms of class I PI3K are ubiquitously expressed in human beings. Therefore, the alterations in this part of PI3K grab more attention from the researchers.

Phosphorylation of Receptor Tyrosine Kinases (RTK) will activate the PI3K. This activated PI3K later helps in the conversion of the phosphoinositide 4,5-biphosphate (PIP2) to phosphatidylinositol triphosphate (PIP3) [3-5]. Further AKT gets activated *via* PDK1 through PIP3. Later PIP3 gets dephosphorylated through the tumor suppressor gene called PTEN to PIP2. However, the complete activation of AKT is only possible through the participation of complex 2 of the mammalian target of rapamycin (mTOR C2).

mTOR is a serine-threonine protein kinase belonging to the PI3K family. This was identified first during the test for the resistance of the immunosuppressant drug called Rapamycin in the budding yeast *Saccharomyces cerevisiae*. mTOR C1 and mTOR C2 are the two distinct multiprotein complexes of mTOR. They play a vital role in the cell regulation [17].

Thus, the over-expression of the PI3K/AKT/mTOR pathway leads to the development of OC. The role of the PI3K/AKT/mTOR pathway stands important here because the overexpression of the PI3K pathway, along with the loss of the tumor suppressor gene PTEN, adds to the development of the cancer cells. In Fig. (**1b**), the role of PTEN is demonstrated, where we can note that the loss of this gene will add to the increase in cancer cells. Therefore, the use of PI3K inhibitors is much needed. The PI3K inhibitors inhibit the pathway in the initial stages itself leading to no further progress in the pathway. However, due to the complexity of the pathway, negative feedback was observed, which led to the activation of the PI3K pathway through mTOR. This gave scope for the development of mTOR inhibitors. mTOR inhibitors were designed such that they inhibit the pathway at the later stages after the activation of PI3K and AKT. Due to the toxicity observed, further studies demonstrated that the inhibition of the PI3K/AKT/mTOR pathway could be effective only by the inhibition of all 3 isoforms of PI3K and mTOR simultaneously.

### 2.1 Role of PI3K/AKT/mTOR Pathway in OC

Though the role of the PI3K/AKT/mTOR pathway in the OC is complex, the dysregulation of this pathway increases the tumor formation, migration of cancer cells, and resistance to the current treatment methods like radiotherapy and chemotherapy. It is also reported to form damage to the primordial follicles during oocyte maturation [18].

Several studies have been conducted to know the role of the PI3K/AKT/mTOR signaling pathway in OC. In one of the studies conducted, loss of the PTEN gene was found in OC affected cells. In another study with four cell lines A2008, OVCAR-8, UPN251 and OVCAR-10, the PIK3CA SiRNA showed the highest growth inhibitor effect in A2008 cells [19]. Therefore, a strong correlation of the SiRNA present in the PI3K/AKT/mTOR pathway with the OC cells can be observed.

There are studies that show the inhibition of mTOR helps in the reduction of the migration of the OC cells. Montero *et al.* reported that the inhibition of RICTOR and RAPTOR protein, which are present in mTOR, inhibits the proliferation of the A2780 cell lines [20].

Studies show that the mutations and amplifications of PIK3CA, deletion of PTEN, and mutations in AKT can be observed in patients with OC [21, 22]. So, these studies give evidence that the alterations in the PI3K/AKT/mTOR pathway lead to the development of OC. Being one of the attractive targets for OC, inhibitors of this pathway can also be classified into 4 different classes: PI3K inhibitors, mTOR inhibitors, AKT inhibitors and dual PI3K-mTOR inhibitors [23, 24].

## 3 PI3K INHIBITORS

**Fig. (2):**
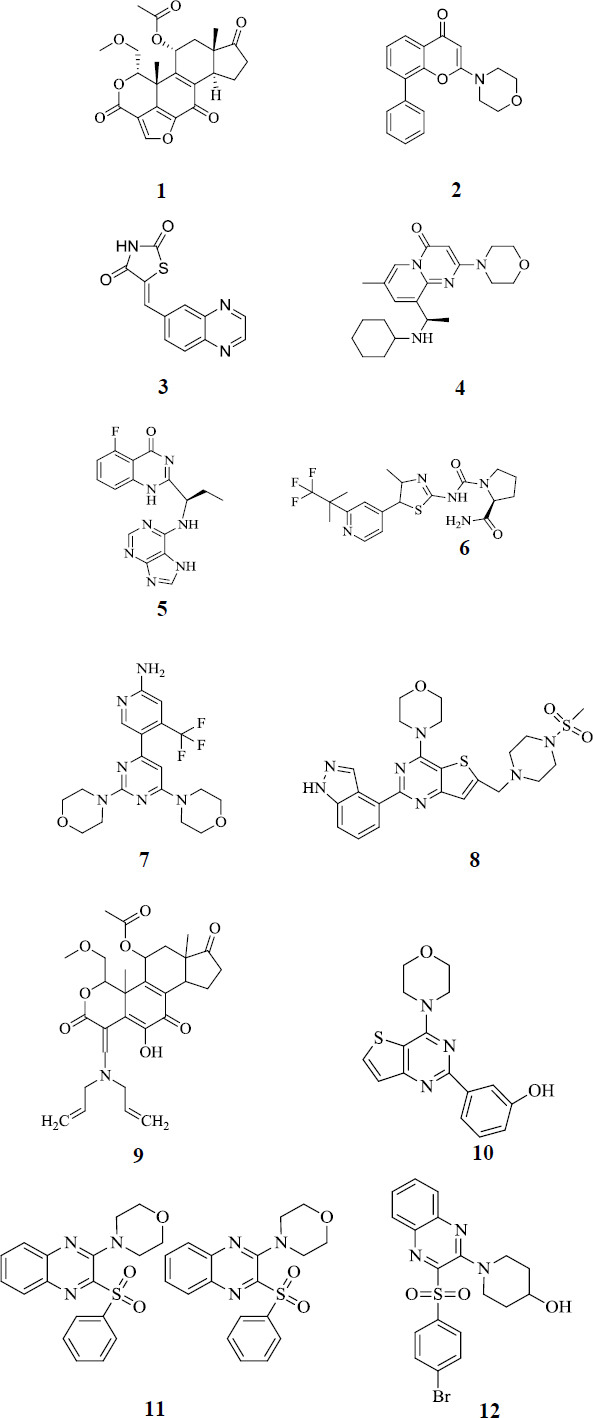
PI3K inhibitor compounds **1-12**.

PI3K inhibitors can be further categorized into two types, namely, isoform selective and pan PI3K inhibitors. Isoform selective inhibitors are the ones that inhibit any one form of PI3K whereas there are inhibitors that can inhibit four isoforms of PI3K together at the same time and are known as Pan PI3K inhibitors. Pan PI3K inhibitors consist of Class I pan PI3K inhibitors, which inhibit all four isoforms (α, β, δ, & γ) of Class I of PI3K. As class I is ubiquitously expressed in mammals, it falls under the interest area of research compared to class II and III. The first Pan-PI3K inhibitors were discovered serendipitously from the mold metabolites (Wortmannin (Compound **1**) and LY294002 (Compound **2**) in the 1990’s) [25, 26]. Studies also show that Wortmannin (IC_50_ = 12 nM) inhibits AKT phosphorylation, as illustrated in Figs. (**2** and **3**). However, Compound **2**, a fungal metabolite, is a competitive inhibitor of the ATP binding site of PI3K that inhibits all the classes of PI3K. It had exhibited 11 and 25 times more activity against P110α/δ unit than P110β/γ unit, respectively. Additionally, it has enhanced the cytotoxic effect of the chemotherapeutic drugs (carboplatin and paclitaxel) in ovarian cancer when tested in the SKOV3 and IGROV-1 cell lines. The poor bioavailability and limited solubility of 1^st^ generation PI3K inhibitors (**1** & **2**) led to the discovery of 2^nd^ generation PI3K inhibitors against the PI3K/AKT/mTOR pathway (Fig **2**).

Abundant research in the field of PI3K inhibitors led to the discovery of selective PI3K inhibitors. The first selective PI3Kγ inhibitor (Compound **3**) is also an ATP competitive inhibitor that fits into the pocket of the ATP enzyme. On the other hand, the first PI3Kβ inhibitor (Compound **4**) has 2 enantiomers, out of which the R-enantiomer is more potent with an IC_50_ value of 0.8µ M (Fig. **2**).

FDA approved PI3Kδ inhibitor (Compound **5**) with an IC_50_ value of 2.5nM in July 2014 and is the first orally potent inhibitor. This FDA-approved PI3Kδ inhibitor has a quinazolin-4-[3H]-one scaffold. Even though the compound and its derivatives show well-versed inhibitory activity, it is followed by the side effects such as diarrhoea and colitis, which may even lead to death. The toxicity profile of compound **5** led to the discovery of novel compounds with good inhibitory activity and fewer side effects (Fig. **2**).

Another FDA-approved PI3K inhibitor (Compound **6**), which selectively inhibits class I PI3K P110 α isoform, was used in the treatment of PIK3CA-related overgrowth spectrum in adults and in children who require systematic therapy [27]. It is also combined with fulvestrant for the treatment of advanced or metastatic breast cancer in postmenopausal women. A combination of Olaparib and compound **6** was used in the preclinical work in the PDX models of ovarian cancer, where it was found that compound **6** inhibits HRR and sensitizes OC models [28]. The use of compound **6** wasshown to increase blood glucose levels, leading to hyperglycemia. A case study conducted by Nicolas *et al.* showed that a patient with no history of diabetes developed hyperglycaemia after the treatment with compound **6** and fulvestrant for breast cancer. Due to the anti-cancer property of compound **6,** it was still used further for the treatment, along with the SGLT2 inhibitor dapagliflozin, which was successful. Still, there are no larger studies related to the importance and advantage of using SGLT2 inhibitors in managing the side effects caused by the PI3K inhibitors [29] (Fig. **2**).

Due to the toxicity observed in the isoform-selective PI3K inhibitors, PAN PI3K inhibitors were considered for further studies to overcome the associated toxicity profile. Substitution of the morpholine moiety in the compounds showed better activity in the trials [30]. Extensive research on the morpholine moiety in PI3K inhibitors led the way to the discovery of compound **7** which could inhibit all four isoforms of class I PI3K. It is classified as a class I pan PI3K inhibitor bearing the pyridinyl pyrimidines scaffold developed by Novartis International AG [31]. Bendell *et al.* conducted the phase 1 trial on compound **7** and exhibited that it binds with the lipid kinase ATP domain, and therefore, it works by inhibiting proliferation, blocking angiogenesis, and promoting apoptosis [32].

Additionally, Muller *et al.* conducted the study to understand the mechanism associated with PI3K inhibition [33]. This study showed that the pan PI3K inhibitor BKM120 has antitumor potential, and is enhanced when used in combination with the MEK1/2 inhibitor. Sun *et al.* showed that the inhibition of PI3K and the SHP2 together will help in inhibiting GAB2 overexpressed ovarian cancer cell lines. Being shown to have good brain permeability independent of the blood barrier efflux transporter, compound **7** shows fast-track status with FDA [34, 35]. Therefore, it can be said that this can be an ideal option to use as a PI3K inhibitor. Even though this has some side effects like hyperglycaemia, respiratory disorder, and some immune system issues, all its advantages make this stand out in the clinical trial [36]. Even though there are still more opportunities to develop a potent inhibitor that has less or can nullify the side effects and treat the disease with limited side effects (Fig. **2**).

Compound **8** is another class I pan PI3K inhibitor belonging to the class of thienopyrimidines [37]. The inhibition of this compound against different isoforms of PI3K can be noted with the IC_50_ values of 3, 33, 3, and 75 nM for α, β, δ, γ, respectively. A study conducted by Raynaud *et al.* showed 98% and 80% growth inhibition in PI3K pathway-activated U87MG glioblastoma and IGROV1 ovarian cancer xenograft model confirmed the antitumor activity of Compound **8** (Fig. **2**) [38].

A synthetic derivative of wortmannin was later discovered (Compound **9**), which was marked as a pan-isoform selective PI3K inhibitor. Compound **9** covalently inhibited the PI3K receptors by forming a covalent bond with lysine 802 in the ATP catalytic site [39]. The efficacy of the compound was tested in phase I clinical trials in combination with docetaxel, where the study was conducted on 43 patients having solid tumors [21, 39]. Amongst all, 5 solid tumor cases belonged to ovarian cancer. Also, studies show that there is a delay in the growth of ovarian cancer cells in the OVCAR3 cell line when treated with compound **9** [40, 41]. This makes the compound more active (Fig. **2**).

Hayakawa *et al.* developed a series of 4-Morpholino-2-phenylquinazoline derivatives, which set a trend for future discoveries regarding the morpholine scaffold (Compound **10**) [42]. Thus, the search for a novel and potent PI3K inhibitor led to the discovery of a morpholine-based (Compound **11**) PI3Kα inhibitor, which had a structural similarity to compound **2** [43]. There was a remarkably strong activity noted against P13Kα by a series of thieno [3,2-d]pyrimidine derivative (IC_50_=2 nM), creating a space as a first selective P13KP110α inhibitor (Fig. **2**).

Later, the studies by Pengwa *et al.* (Compound **12**) shed light on novel 2-aliphatic cyclic amine-3-[aryl sulfonyl] quinoxalines, which was developed from the compound 11 modification [44]. With an IC_50_ value of 0.025µM, compound **12** stood out as the most potent PI3Kα inhibitor. From the reported studies, (Compounds **2, 11, 12**) a novel piperazinyl quinoxaline derivative derived as the potent PI3K alpha isoform inhibitors. Replacement of the 4-morpholino group with the 4-carbamoylpiperidin-1-yl group at the 2^nd^ position of the quinoxaline had a similarity with compound **12** and showed an interesting *in vitro* activity. This study concluded that the piperazinyl quinoxaline derivatives can be used as PI3K inhibitors for cancer therapy (Fig. **2**).

### 3.1 Discussion of PI3K Inhibitors

As discussed earlier, the compound **1** and **2** during the clinical trials, were found to be less bioavailable. These studies resulted in the hunt for other PI3K inhibitors. The search led to the discovery of the 2 ^nd^ generation PI3K inhibitors.

A study conducted by Li *et al.* for breast cancer using compound **3 **showed that the inhibition of PI3K gamma reduces the growth of tumor cells [45]. Considering the fact that PI3Kγ inhibition could reduce tumor growth in other cancers, there was not much evidence that showed the inhibition of PI3Kγ reducing ovarian cancer cells. However, there were studies reported related to OC showing the inhibition of PI3K-alpha [43, 46].

Another common challenge observed in treating patients through inhibition of PI3K is that of the toxicity from on-target and off-target effects [47, 48]. Commonly reported side effects were hyperglycemia, diarrhea, fatigue, and nausea. Some less common side effects like hypertension and central nervous system problems were noted by compound **7**.

There are many Pan PI3K and isoform-selective PI3K inhibitors that are also in trials for OC but due to their high toxicity and less efficacy, there is still research going on in this field. Though the side effects can be taken care of by careful observation of patients, there are no such reports showing the drugs with zero or nullified side effects as PI3K inhibitors. Further, the development of the mTOR inhibitors also paved the way for the advancement in the field of inhibitors of the PI3K/AKT/mTOR pathway.

## 4 mTOR INHIBITORS

A mammalian Target of Rapamycin (mTOR) was discovered following the discovery of the Rapamycin. A soil sample from Easter Island collected in 1965 had a soil bacterium *Streptomyces hygroscopius*, which later paved the way for the discovery of rapamycin, a secondary metabolite, in 1970 [49, 50]. A study by Heitman *et al.* in 1991 led to the discovery of TOR [49].

## 5 FIRST-GENERATION mTOR INHIBITORS

mTOR was discovered based on its FRBP-rapamycin binding property in 1994 [51, 52]. This mammalian target of rapamycin has control over the protein, ribosomal biosynthesis, transcription, translation, and cell growth [53, 54]. Therefore, this is also termed the “central controller of cell growth.” Human mTOR has a property wherein it can bind with different proteins and can form two different types of polyprotein complexes-mTORC1 and mTORC2. mTORC1 has 5 subunits whereas mTORC2 has 6 subunits. Out of all the subunits, raptor and rictor are the distinct subunits present in mTORC1 and mTORC2, respectively [55, 56]. Overexpression or the over-activation of mTOR will lead to abnormal diseases. Over-expression of PI3K/AKT may lead to the over-activation of mTOR, which in turn will end up in the development of cancer cells or other diseases. So, inhibition of this mTOR pathway is the most necessary need at this time. The discovery of mTOR inhibitors started in different stages, and the first generation was the antibiotic-allosteric mTOR inhibitors. Out of the first-generation inhibitors, compound **13** was first discovered, and it is an allosteric inhibitor [57]. Later, based on this, the studies were developed, which led to the discovery of derivatives of compound **13**. This compound **13** was known to be directly inhibiting mTORC1 and simultaneously inhibiting mTORC2. Even though the half-life of compound **13** is 50 hrs due to its poor water solubility and stability, its biological utilization is low, and this, in turn, leads to limited clinical applications. Therefore, the search further was based on the modifications of this moiety itself [58].

**Fig. (3):**
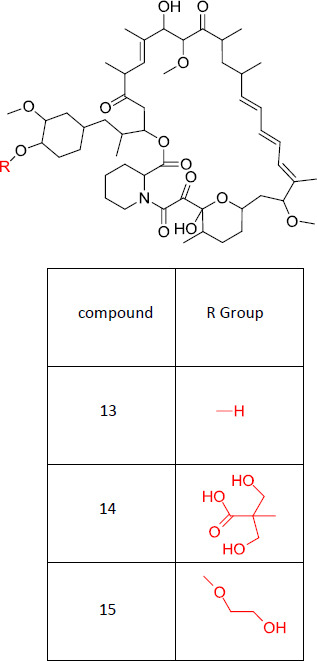
First-generation mTOR inhibitors.

The first derivative of compound **13** was developed by Wyeth by the esterification of one of the hydroxyl groups present. This developed compound (Compound **14**) was found to be more stable than the earlier discovered compound [59]. Due to the degradation of compound **14**, it was only used for intravenous administration [60]. Still, the need for a compound that would show better activity led to the discovery of compound **15**, which again is the derivative of compound **13** prepared by esterification by Novartis. There was a limitation in this case, as it showed toxicity in a few patients (Fig. **3**) [59].

## 6 SECOND-GENERATION mTOR INHIBITORS

Inhibition of mTORC1 or mTORC2 will, however have the chances of activating AKT in the pathway, and hence, there was the need for dual mTOR inhibitors. This further paved the way for the development of second-generation inhibitors. Most of the developed second-generation inhibitors were ATP competitive and PI3K/mTOR dual inhibitors. They were found to bind directly on the ATP site of PI3K/mTOR. However a majority of them were found to be mTOR selective. This leads to the conclusion that the second-generation mTOR inhibitors are better mTORC1/mTORC2 dual inhibitors or selective to the mTOR pathway. They were proved to have lower IC_50_ values against mTOR than PI3K. The special feature of the second- generation compounds was that their discovery revolved around the same scaffold. The alterations and deletion of some groups on the same scaffold brought in wide changes in their activity. Therefore, our attempt here is to show the activity based on the structure-activity relationship according to the scaffold.

## 7 STRUCTURAL ACTIVITY RELATIONSHIP OF SECOND-GENERATION mTOR INHIBITORS WITH DIFFERENT SCAFFOLDS

### 7.1 Pyrido [2,3-d]Pyrimidines

**Fig. (4):**
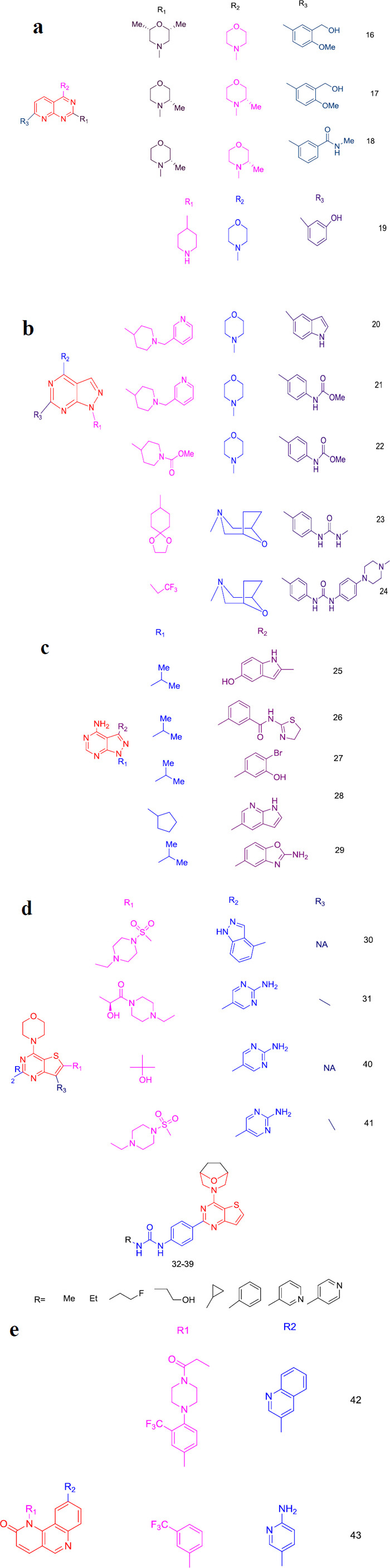
(**a**) Pyrido[2,3-d]pyrimidine derivatives. (**b**) Pyrazolo[3,4-d]pyrimidines derivatives. (**c**) 4-Aminopyrazolo[3,4-d]pyrimidines derivatives. (**d**) Thieno[3,2-d]pyrimidines derivatives. (**e**) Benzo [h]1,6-naphthyridin-2-ones derivatives. Second-generation mTOR inhibitors mentioned in (**a-e**).

Pyrido [2,3-d]pyrimidines scaffold was used to develop a class of three compounds that showed structural similarity with a slight alteration that led to a great difference in their inhibition activity against mTOR. Compounds **16-18** share a common structure with a morpholine or methyl morpholine substituent in the second position and a 3-/4-phenyl substituent on the pyridine ring (Fig. **4**).

Compound **16** has 2 morpholines, out of which one is a di-methyl substituted morpholine at the second position. It was shown to have mTORC1 and mTORC2 inhibition action with an IC_50_ value of 50 nM [61]. There is no evidence of this showing PI3K inhibition. Compound **16** showed a better dephosphorylation when compared with compound **13** to 4E-BPI. This makes the compound an ATP-competitive mTOR inhibitor [61].

Alterations on the substituents present in compound **16** led to the discovery of another compound with an IC_50_ value of 0.8 nM. This compound **17** has both methyl-substituted morpholine present at the R_1_ and R_2_ positions of the scaffold. Leading to the cell death compound **17** showed a better result in a study possessing HCC cells by inducing AMPK activation and autophagy [62, 63]. Compound **17**, when used against Hep-2 cell lines, showed that it has antiproliferation and apoptosis induction activity. Unfortunately, due to its unsustainability in clinical trials and increase in transaminases it lead to dysfunction of the liver cells and, ultimately, liver damage occurred. This left me with no other option than to search for a better alternative.

Another alteration in the scaffold was executed by replacing the hydroxymethyl group with the methyl carbonyl group to obtain compound **18**, which showed a reduced increase of transaminase compared to compound **17** [63]. Compound **18** showed good activity in the solid tumor-based phase I study. Further studies showed that this is inferior to compound **15** and leads to rictor amplifications which further makes way for many other difficulties. Therefore, keeping these limitations in mind, compound **18** was terminated in 2018 [64, 65].

Comparing the activity of compounds **16-18** one can see that they showed good mTOR inhibition activity, but still, due to their toxicity and side effects, they couldn’t lead to be the heroes in the mTOR inhibitors category. This shows that there are still scopes for alterations in this scaffold by which the toxicity can be reduced and a compound with better activity can be discovered.

### 7.2 Pyrazolo [3,4-d]Pyrimidines

**Fig. (5):**
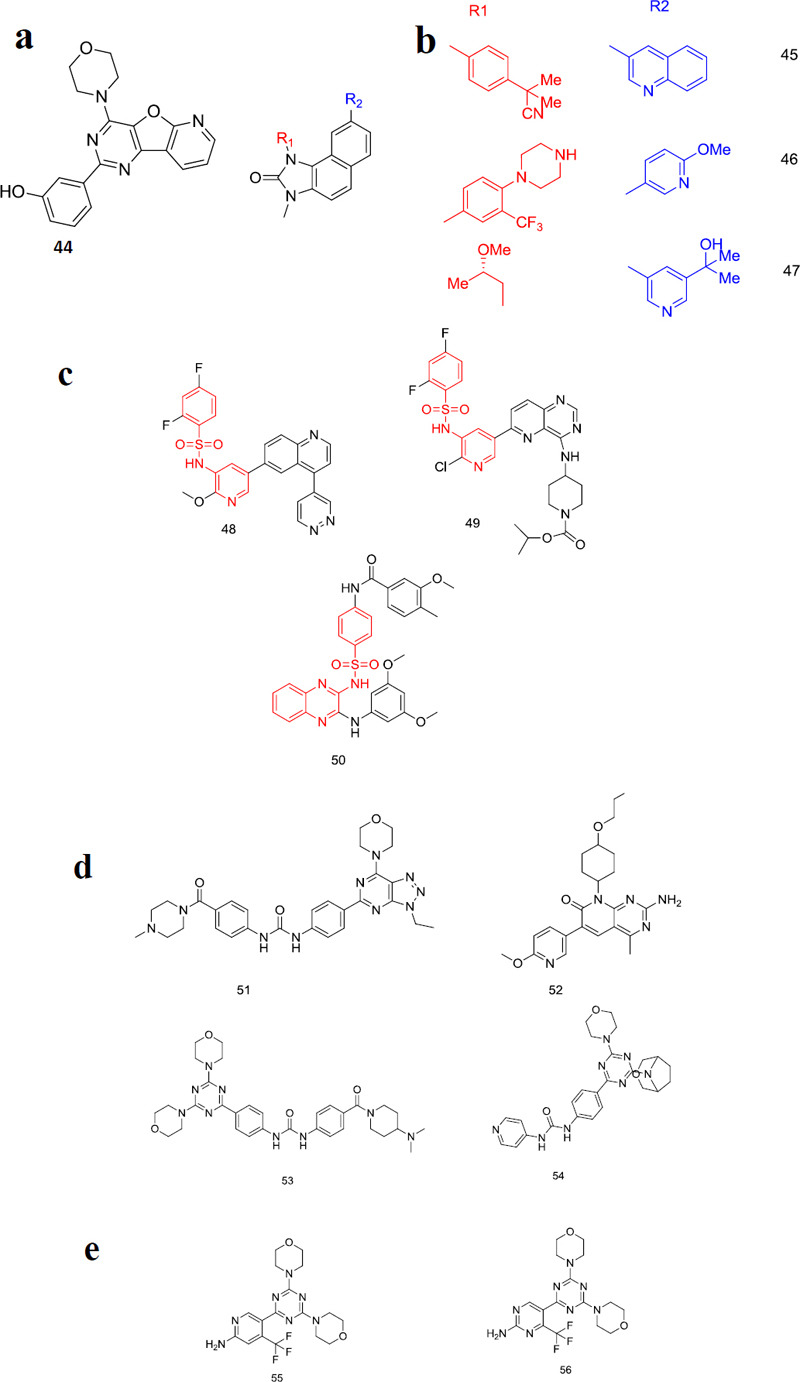
(**a**) First dual PI3K/mTOR. (**b**) Dual PI3K/mTOR inhibitors. (**c**) Sulphonamide dual PI3K/mTOR inhibitors. (**d**) Dual PI3K/mTOR inhibitor compounds **51** and **52**. (**e**) Dual PI3K/mTOR inhibitor compounds **53-56**. Dual PI3K/mTOR inhibitors with different scaffolds mentioned in (**a**-**e**).

Wyeth developed a group of compounds sharing the same scaffold Pyrazolo [3,4-d]pyrimidines with slight alterations in the 1^st^, 4^th^ and 6^th^ positions. Compounds **19** to **24** have Pyrazolo [3,4-d]pyrimidines scaffold with a morpholine or derivative of morpholine present at the 4^th^ position (R_2_ position) [66]. Table **1** shows the IC_50_ values of each compound against mTOR. Out of all the compounds, compound **24** shows agreeable activity against mTOR with 0.6 nM IC_50_ value whereas 150 nM against PI3K which is a drastic increase of 250 folds. This difference makes this compound an outstanding mTOR inhibitor [67]. Compound **23** inhibits both C1 and C2 complexes of mTOR and causes tumor regression in a study with mouse xenograft models. This showed that compound **23** is more selective towards mTOR than PI3K about 5000 times (Fig. **5**) [68]. Selectivity can be seen due to the strong G1 arrest in both the CCI-779 sensitive and CCI-779 resistant cells at a particular concentration that does not target the P-AKT.

### 7.3 4-Aminopyrazolo [3,4-d]Pyrimidines

The same scaffold replacement of the morpholine ring with the amino group in the second position gave rise to derivatives that showed good inhibitory activity against mTOR. These derivatives (Compounds **25** to **29**) do not have an attachment at the R_3_ position, unlike those seen in compounds **19** to **24** (Fig. **4**) [69]. The IC_50_ values of these compounds against mTOR are depicted in Table **2**.

### 7.4 Thieno [3,2-d]Pyrimidines

As the search for the highly selective and potent mTOR inhibitors continued, studies with the substitution of scaffold thieno [3,2-d]pyrimidines at R_1_, R_2_, and R_3_ positions came forward. The common features of these derivatives were that the thieno group and the morpholine derivatives were attached to the pyrimidine ring. Also, the substitution of methyl on the R_3_ position of the scaffold can be noted in some derivatives, which gives a clear idea that if any large group is attached to this position, it may lead to a huge difference in the activity of that compound. Compound **30,** being a derivative of this scaffold, gave a starting point for further discoveries as it showed an IC_50_ value of 3nM for PI3K whereas 150nM for mTOR [70]. Later, modification of the attached groups gave compound **31,** which had an attachment of methyl group at the R_3_ position. Compound **31** showed the Ki value of 17 nM for mTOR and IC_50_ values of 5 nM, 27 nM, 7 nM, 14 nM for PI3Kα, β, δ, γ respectively [71]. This replacement of the functional groups on the compound showed that it has a better inhibitory action compared to compound **30**. Studies showed that compound **31** inhibits certain breast cancer, pancreatic cancer, and colon cancer cell lines [72, 73]. It has also shown to inhibit prostate cancer and melanoma cell lines. Though the activity was poor it had a better result when compared with the compound **30**. Compound **31** also passed the phase I study, showing good inhibitory action, but it was not best compared with compound **15** as it led to the blockade of the whole pathway. Verheijen *et al.* continued the same study by making some changes in the substitution on the scaffold [74]. They replaced the morpholine group at the 4^th^ position with the 8-oxo-3-azabicyclo[3.2.^1^]octan-3-yl and also the 2-aryl group with 4-ureidophenyl which showed a drastic change in the inhibitory activity against PI3K and mTOR. The IC_50_ values of these compounds (Compounds **32**-**39**) are depicted in Table **3** [74]. Out of those compounds, compounds **33**, **34**, and **36,** when calculated, showed a selectivity of over 1000 to mTOR (selectivity = IC_50_ of PI3Kα/IC_50_ of mTOR) (Fig. **4**).

Further modifications were madeagain at the 2^nd^ position of the thieno [3,2-d]pyrimidine scaffold, giving compounds **40** and **41** [75]. IC_50_ value of compound 40 is 30 nM against mTOR and 3.4 nM against PI3Kα whereas compound 41 has a Ki value of 21 nM against mTOR and 4 nM against PI3K [76].

### 7.5 Benzo [h]1,6-naphthyridin-2-ones

Quinoline condensed with pyridine gives a scaffold Benzo[h]1,6-naphthyridin-2-ones, which was the focus scaffold for the mTOR inhibitor. A study with the substitution at the 1^st^ and 9^th^ position of the ring gave compounds **42** and **43**. Liu *et al.* studied compound **42** and found that the IC_50_ value is 0.29 nM against complex 1 and 2 nM against complex 2 of mTOR [77]. The half-life of this compound was reported to be 4 minutes. Recent reports show the intervention of compound **42** with mTOR signaling in the insular cortex that eases neuropathic pain [78]. Even though it showed a good inhibitory activity, its unsatisfactory stability and poor oral bioavailability did not allow it to proceed for the *in-vivo* studies. As the inhibition was good, researchers then thought of altering the substitutions. Therefore, the replacement of the double-ring substitution at the R2 position with a single-ring structure and with similar changes in the R1 position gave compound **43** [79]. These substitutions were quite successful as they could increase the half-life to 17.7 minutes and increase the stability [79]. IC_50_ of 37.1 nM against the mTORC1 and 25 nM against mTOR was noted. Presently, this compound is in preclinical studies on thyroid, liver, colorectal and breast cancer (Fig. **4**) [80].

## 8 THIRD-GENERATION mTOR INHIBITORS

Rapalinks are the third-generation mTOR inhibitors. They are designed uniquely, which consist of 3 parts: one ATP inhibitor, a linker and rapalog. Rapalogs are the derivatives of rapamycin with ATP inhibitors that are attached to certain carbon atoms called linkers. When these Rapalinks are used as mTOR inhibitors, the ATP inhibitor will interact with the ATP binding site, and meanwhile, rapamycin targets the mTOR, which makes them more effective and specific.

The first rapalinks share a common ATP binding site (Compound **29**) and rapamycin but are connected with different linkers. Linkers help to connect the 1-N atom of compound **29** and the 42-O atom of rapamycin. They have 39, 36 and 11 carbon atoms in their linkers, respectively. Out of the three, rapalink with the least carbon atom is the smallest and less effective compared to others.

### 8.1 Discussion of mTOR Inhibitors

Inhibition of mTOR plays a crucial role in inhibiting the PI3K/AKT/mTOR pathway. If we can compare all the inhibitors against mTOR which were discussed above, we can note that they share a common feature of having a morpholine moiety or its derivatives. This resemblance can also be seen in the PI3K inhibitors, as delineated above. This gives us a clear conclusion that along with the main scaffold, the presence of the morpholine moiety also adds to better inhibition. Therefore, researchers for many years have been trying to build a mTOR inhibitor with the presence of morpholine.

Inhibition of the mTOR is a wide area of research. There are many reasons for it to stand out and be ineffective in cancer studies. Research shows that one of the main reasons is that the inhibition of mTOR alone may trigger the upstream loops, which may pave the way for the survival of cancer cells, hence not serving our purpose [81, 82]. Also, studies show that the second-generation inhibitor Torin1 is more toxic to islet cells. Although, inhibition of mTOR may help in protecting the cancer cells, one should also keep in mind the negative effects of the same. As a solution, one can think of inhibiting all 4 isoforms of PI3K, mTORC1 and C2 [22, 83]. This will act as a preventive measure from upstream activation. Also, these dual inhibitors were proved to have less toxicity compared to the pan PI3K’s.

## 9 PI3K-mTOR DUAL INHIBITORS

PI3K/AKT/mTOR is the most crucial pathway in cancer inhibition. As discussed earlier many inhibitors target this pathway either by PI3K or mTOR inhibition alone. However, chemoresistance brings limitations to the chemo drugs. To overcome this difficulty, there is a need for drugs that inhibit the activity of pathways and increase sensitivity to the chemo drug. The challenge while targeting the PI3K/AKT/mTOR is to balance the interactions between the PI3K. AKT, mTOR because when mTOR is inhibited, it activates AKT and PI3K. This will reduce the effect of the designed small-molecule drug. So this paved the way for the development of the dual inhibitors. Inhibition of PI3K/mTOR together will reduce the cancer cell proliferation and induce apoptosis. Gazi *et al.* carried out a study to find the effect of the single and dual-targeted inhibitors where they applied single and dual-targeted inhibitors to patients with children’s recurrent acute lymphoblastic leukemia [84]. This study revealed that the dual inhibitors reduced the cell viability and induced apoptosis when compared with the single-targeted inhibitors. On the other hand, the “double-edged sword” autophagy that helps the survival of the cancer cells and at the same time induces cell death is regulated by the PI3K/AKT/mTOR pathway. It controls autophagy by phosphorylating various substrates, which are the downstream effectors of mTORC1. As discussed earlier, if we inhibit mTOR alone the rest part of the pathway might receive feedback to activate. Hence, instead of developing single targeted molecules for the pathway, it is better to develop a dual-targeting drug. Having said that dual PI3K-mTOR inhibitors have less toxicity and common symptoms of side effects like nausea, fatigue, vomiting *etc.*, there are very limited dual PI3K/mTOR inhibitors that have advanced the clinical trials for cancer. Out of all the PI3K/mTOR inhibitors in the clinical trials there are only a few inhibitors that can be used for OC. Astella developed the first dual PI3K/mTOR dual inhibitor with morpholino quinazoline derivative through high throughput screening (Compound **44**) [85]. The IC_50_ value of compound **44** was found to be 3.6 nM for PI3Kα and 3.0 nM for mTOR [86]. Even though compound **44** showed good inhibitory activity on tumor cells, it showed poor drug properties (Fig. **5**).

Another oral PI3K/mTOR dual inhibitor developed by Novartis, compound **45**, showed IC_50_ value 20.7 nM against mTOR and 4 nM, 75 nM, 7 nM, and 5 nM against α, β, δ, γ respectively [87]. Compound 45 further showed good inhibitory activity against breast cancer when compared with other PI3K inhibitors [88, 89]. Compound **45**, when used for the treatment of mTOR inhibitor-naïve patients with advanced pancreatic neuroendocrine tumors (pNET), failed to show greater efficacy than compound **15** and showed a poor tolerability profile. In the phase II study, toxicity was found in a few patients, but still, very less patients showed good activity, which left space for further study using this moiety [90, 91].

Novartis modified the compound **45** and developed another oral drug that showed cytotoxic activity in both normoxic and hypoxic HCC cells, which suggested that it can be used for HCC-targeted cancer treatment (Compound **46**) [92].

A water-soluble derivative of the same scaffold was developed, which was used as an oral drug against the PI3K/mTOR pathway [93, 94]. It showed IC_50_ values of 165nM against mTOR and 6.07, 77.6, 38, and 23.8 nM against α, β, δ, γ, respectively. Studies showed that developed compound **47** had good anti-tumor activity against glioma cells, skin carcinoma and oesophageal adenocarcinoma rat models [95]. Phase I study showed that this compound has a good safety profile [95]. Phase II studies of this compound showed modest single-agent activity and a manageable safety profile in advanced endometrial cancer (Fig. **5**) [96].

The search of different scaffolds led to the selection of another scaffold, benzene sulphonamide, where the benzene ring has an attached sulfonyl group where the N of the sulfonyl group is connected either with the pyridine or other N-containing heterocycle compounds. Compound **48** with the same pyridine benzene sulphonamide showed a Ki value of 0.18 nM, 0.3 nM, and 0.019-0.13 nM, against mTORC1, mTORC2, and PI3K respectively [97, 98]. Inhibition of nasopharyngeal carcinoma and improvement in radiotherapy can be noted from the previous studies [98, 99].

CMG pharmaceuticals used the same scaffold to develop compound **49**, which is cytotoxic in chemo-resistant OC cells [100, 101]. This compound showed inhibition of HCC cell proliferation and tumorigenesis when used in combination with sorafenib. Therefore, these studies direct the use of compound **49** as a new therapeutic strategy for OC and HCC [102].

Exelixis developed another compound, which was modified from a single targeted PI3K inhibitor. Compound **50** with quinoxaline benzene sulphonamide scaffold showed better activity on prostate cancer cells in comparison with compound **13** [103, 104].

Another PI3K/mTOR dual inhibitor with triazolo [4,5-d]pyrimidine structure, compound **51** showed IC_50_ values of 1.7 nM, 1.4 nM, 9.2 nM against mTOR, PI3Kα and PI3Kγ respectively [105]. Hu *et al.* conducted a study on cisplatin-sensitive A2780 and cisplatin-resistant SKOV3 ovarian cancer cell lines and found the dual inhibition of compound **51** [105]. This study also contained a comparison of the activity of one PI3K inhibitor and compound **51**, which showed that compound **51** showed good activity by increasing cell viability, proliferation, and apoptosis.

Pyrido [2,3-d]pyrimidine scaffold was used for the development of compound **52**, which again showed good inhibitory activity and IC_50_ values of 1.6-2.1 nM against P13K α-γ and Ki values of 16 ± 4.0nM against mTOR.

Recently, the use of 1,3,5-triazine scaffolds has been observed in the designing of PI3K/mTOR dual inhibitors. 2,4-dimorpholinyl-1,3,5-triazine scaffold with the piperidine ring substituted with benzoyl group at the 1^st^ position was used for the synthesis of compound **53** which showed the IC_50_ values of 16 nM against mTOR, 0.4 nM PI3Kα and with a half-life of 4.4 hrs [106]. In a Phase II study done on patients with recurrent endometrial cancer, compound **53** demonstrated acceptable tolerability and good activity [107, 108].

Modification of scaffold used in compound **53** with the two-carbon bridge on R_2_ morpholine and replacement of benzamide part present at the end of R_3_ carbon with pyridine-4-yl reduced the molecular weight of the compound and IC_50_ value reduced to 0.42 nM against mTOR, and 8 nM against PI3Kα [109, 110].

Another dual inhibitor, a pyridine derivative with 1,3,5-triazine, showed IC_50_ values of 89 nM against mTOR and 33 nM against PI3Kα (Compound **55**) [111]. Compound **55** reached phase II trials. Later, Borsari *et al.* modified compound **55** and the designed compound was a pyrimidine derivative (Compound **56**) with Ki values of 33 nM and 2.2nM against mTOR and PI3K, respectively (Fig. **5**) [112].

Thus, we can see that there are very limited inhibitors that target both mTOR and PI3K but there are hardly few inhibitors that are presently in use for OC. Present studies show the use of 1,3,5-triaizne with the attachment of two morpholine which makes the structure planar [113].

## 10 RECENT TRENDS IN PI3K/AKT/mTOR INHIBITORS FOR OC

In this review article, we discuss the literature regarding the trial and error faced while designing inhibitors for various cancers. Our main aim of this review is to find a potent scaffold to treat OC. Through the studies over the years, we can see that the molecular targeted therapy through this pathway was with chemo or radiotherapy that promised positive treatment for the patient. The drugs in the clinical trials have only reached the initial trials or have been rejected in the late- phase of clinical trials due to toxicity. Through our literature survey, we have found that the inhibition of four PI3K isoforms with 2 mTOR classes together can help us overcome the toxicity and side effects present in the current treatment methods. However, there are only a limited number of drugs that have entered the clinical trials as dual PI3K-mTOR inhibitors for OC. A few examples of the drugs that showed anti-tumor activity in pre-clinical studies were compounds **52, 53, 55**. Compound **52** showed a reduction in tumor volume in PIK3CA mutant SKOV3 xenograft models. CMG002, compound **49**, also demonstrated a decrease in the OC cell growth in the xenograft models [101]. Another PI3K inhibitor that is currently in trend as a dual inhibitor (Compound **53**) is Gedatolisib. It contains a triazine scaffold with the 2 morpholine rings attached. Gedatolisib is currently in trials with Crizotinib against OC cell lines [114, 115].

Other studies show that the compound GSK458 and Samotolisib showed positively good inhibitory activity for OC in phase I of clinical trials [116]. Still the evidence for a dual PI3K/mTOR inhibitor that can treat OC with zero side effect is not found. Therefore, through this review, we carefully observed the change in scaffold for the inhibitors over time and found that the current trend is in the use of triazine as the scaffold. This scaffold surprisingly showed great inhibitory activity and few compounds have entered the early phase of clinical trials. Therefore, more preference for a triazine ring with morpholine substitution can be thought of while designing of PI3K-mTOR dual inhibitor for OC.

## CONCLUSION

Several research works are in progress in inhibiting the PI3K/AKT/mTOR pathway, as it is one of the most altered pathways in OC. Disappointment is that there are still no FDA-approved drugs in the market that target this pathway. Comparing the individual PI3K and mTOR inhibitors with dual inhibitors, one can note that the effectiveness is much higher in dual inhibitors. Hence, recent studies focus more on inhibiting the PI3K and mTOR together as they will not show any negative regulation or upstream regulation. Also, studies revealed that dual targeting will reduce toxicity, too. As the tendency seen in the use of 1,3,5-triazine scaffold, this has given scope for further development and designing using this scaffold. Further, the use of other ATP inhibitors and mTOR inhibitors in the synthesis of Rapalinks gives more scope to obtain many molecules that can inhibit mTOR effectively. Most of the designed drugs that are present have both positive and negative effects. Knowing the effectiveness of a particular scaffold that is crucial for PI3K/mTOR inhibition and working on that for developing an effective PI3K/mTOR dual inhibition will pave the way for better drugs in the future. The use of these inhibitors with chemotherapy or targeted dosage forms may lead to much more advancement in the treatment of OC and a new topic in research in the future.

## Figures and Tables

**Table 1:** IC_50_ values of pyrazolo[3,4-d]pyrimidines derivatives.

**Compounds**	**IC_50_ (nM)**
**19**	220
**20**	9
**21**	7
**22**	5
**23**	0.19 ± 0.07
**24**	0.6

**Table 2:** IC_50_ values of 4-aminopyrazolo[3,4-d]pyrimidines derivatives.

**Compounds**	**IC_50_ (nM)**
**25**	8
**26**	80
**27**	72
**28**	10

**Table 3:** IC_50_ values of thieno[3,2-d]pyrimidines derivatives.

**Compounds**	**PI3K (nM)**	**mTOR (nM)**
**32**	423	0.9±0.1
**33**	1262	0.7±0.1
**34**	825	0.7±0.2
**35**	324	0.34±0.03
**36**	1359	0.68±0.05
**37**	82	1.25±0.07
**38**	119	0.29±0.01
**39**	80	0.44±0.07

## LIST OF ABBREVIATIONS

OC: Ovarian Cancer
RTK: Phosphorylation of Receptor Tyrosine Kina
MTR: Mammalian Target of Rapamyci
